# RETRACTION NOTICE: Possible treatment for cutaneous lichen planus: An in vitro anti-inflammatory role of Angelica polysaccharide in human keratinocytes HaCaT

**DOI:** 10.1177/20587384211040395

**Published:** 2021-09-02

At the request of the Journal Editor(s) the following article has been retracted.

Wang J, Chen G, Shi T, Wang Y, Guan C (2019) Possible treatment for cutaneous lichen planus: An in vitro anti-inflammatory role of Angelica polysaccharide in human keratinocytes HaCaT. *International Journal of Immunopathology and Pharmacology* 33: 1-12. DOI: 10.1177/2058738418821837.

Significant similarities between this paper and a number of other papers have been identified, as well as indication of image and data manipulation suggestive of the use of a “paper mill”. These papers share common structures and wording yet do not share any common authors.

The authors did not provide a sufficient response or evidence to refute these claims. As a result, the scientific accuracy of this paper is not reliable.

Adhering to the international guidelines established by the Committee on Publication Ethics, the Journal has determined these are grounds for retraction.

